# Reduction of Influence of the High-Frequency Noise on the Results of Surface Topography Measurements

**DOI:** 10.3390/ma14020333

**Published:** 2021-01-11

**Authors:** Przemysław Podulka

**Affiliations:** Faculty of Mechanical Engineering and Aeronautics, Rzeszow University of Technology, Powstancow Warszawy 8 Street, 35-959 Rzeszów, Poland; p.podulka@prz.edu.pl; Tel.: +48-17-743-2537

**Keywords:** surface texture, surface topography, surface topography measurement, measurement noise detection, plateau honing, grinding, turning, cylinder liner, piton skirt, oil pocket, isotropic texture

## Abstract

The influence of errors in the processes of detection and then reduction of surface topography measurement noise is of great importance; many research papers are concerned with the definition of this type of measurement error. This paper presents the influence of high-frequency measurement noise, defined for various types of surface textures, e.g., two-process plateau-honed, turned, ground, or isotropic. Procedures for the processing of raw measured data as a detection of the high-frequency errors from the results of surface topography measurements were proposed and verified (compared) according to the commonly used (available in the commercial software of the measuring equipment) algorithms. It was assumed that commonly used noise-separation algorithms did not always provide consistent results for two process textures with the valley-extraction analysis; as a result, some free-of-dimple (part of the analyzed detail where dimples do not exist) areas were not carefully considered. Moreover, the influence of measured data processing errors on surface topography parameter calculation was not comprehensively studied with high-frequency measurement noise assessments. It was assumed that the application of the Wavelet Noise Extraction Procedure (WNEP) might be exceedingly valuable when the reduction of a disparate range of measured frequencies (measurement noise) was carefully considered.

## 1. Introduction

Precise surface topography measurement and data analysis are reasonably crucial to evaluate the mechanical behavior of “engineering” surfaces [1] and are often an integral part of process control [2]. Surface topography is of great importance, as an example, when the oil consumption mainly caused by the engine’s piston–piston rings cylinder liner system is taken into consideration. The tribological behavior of a piston ring–cylinder liner frictional pair was improved by liner surface texturing [3] with the characterization of the shape of height distribution [4]. Many measurement systems are integrated into the manufacturing process to provide in situ measurement and real-time feedback, some with non-contact approaches with measurement of features generated by a robotized surface finishing system. Therefore, measurement and analysis of surface topography as a fingerprint of the manufacturing process is completely justified. In fact, very precise surface texture measurement equipment may not lead to receiving an accurate result when an appropriate method for the processing of measured data is not provided.

In many previous extensive measurement studies, the various methods (stylus or optical) for surface topography measurement were compared [5,6], e.g., confocal microscopy, coherence scanning interferometry, atomic force microscopy [7], focus variation microscopy, etc. It was also tested if the two results could be integrated to enhance the behavior and performance consideration of complex surface topography measurement and analysis.

Non-contact methods are very popular in surface topography measurements, especially because of its time of measuring, where optical methods [8] are much faster than a stylus. One of them is Scanning White-Light Interferometry (SWLI). This technique has increased in importance for both research (development) and manufacturing quality control [9]. This type of measurement is often presently called “probably the most useful optical instrument” for measuring surfaces, films, and coatings [10] especially because of being susceptible to a skewing effect when the amplitude is less than the coherence length of the light source. Moreover, various types of environmental disturbance can introduce noise in different bandwidths, and noise can be reduced by averaging signals over a longer duration [11]. However, optical instruments applied for measurements of areal topographies can be particularly sensitive to noise presence when scanning is required. Furthermore, the noise has different sources, including those internally generated and external sources from the environment [12]. It was also assumed that profilometer after thermal stabilization shows 90% less noise than in the case of an unstable profilometer when thermal sources of errors in surface texture imaging were considered [13]. Evaluation of every single contributor is not always achievable for some instruments. Nevertheless, it can be quite possible to assess the noise added to the output when the regular use of the device occurs.

In general, noise in surface topography analysis can be divided into scattering noise [14], background noise [15], measurement or instrument noise [16], white noise [17], noise-like spikes or simply outliers [18], data processing noise errors [19], static noise [20], and other noise-like errors [21,22]. Additionally, typical stylus errors can be presented with the influence of mechanical filtering of the stylus tip, skid in the measurement uncertainty [23], stylus flight on the parameters, or errors caused by surface replication. According to the above statement, noise can be highly correlated with the signal or can be in a different frequency band, in which case it is not correlated [24]. The background noise for laser confocal sensor metrology is primarily induced by laser power saturation, stray light, and sharp-edges’ scattering effect [25]. To simplify, the measurement noise can be defined as the noise added to the output signal occurring during the normal use of measuring instrument [26]. Measurement noise is a “dynamic phenomenon”, which is affected by both the motion of the drive unit and instrument internal noise or environmental disturbances [12]. To create a standard reference frame for describing measurement noise, it is necessary to describe it along with the associated measurement bandwidth, expressing it in terms of noise equivalent height, in nm, divided by the square root of the data acquisition rate in height points per second [11]. Definition of noise with the high-frequency domain is still one of the most daunting tasks, although filtering of the surface was performed with removal of high-frequency noise and long-scale waviness/form [27] to maximize the examined measurement bandwidth [28]. Evaluations of measurement noise, which is dependent on the tilt of the analyzed surface, and topography repeatability in very rough surface measurements also seem to be exceptionally complex. In the literature, valuable and detailed information on this type of measurement errors is quite rare; this issue is out of the scope of plenty of scientific papers, correspondingly. To the above, static noise and autofocus repeatability are considered for the contribution separation of the drive unit and that of environmental disturbances. Evaluating of measurement noise was proposed with the subtraction method or the averaging method [29]; both require repeated measurement of a calibrated optical flat. Otherwise, in SWLI, the skewing effect leads to spike-like noise errors in the profile data near steep edges [30]. Errors of non-measured points occurrence, one of the most critical problems in optical surface texture assessments, were also studied with different intensity of measurement light [31]. ISO 25178 neglects the noise in the x- and y-directions. Consequently, the amplification, linearity, and noise in this defined z-direction are primary aspects [32], and researchers have suggested defining the x-direction with y-direction noises.

Contrary to the existing noise-separation methods, in this paper, power spectral density (PSD) was also proposed for characterizing high-frequency measurement noise. This type of analysis was applied previously when the S-F (surface derived from the S-F surface by removing the large-scale components using an L-filter) [33] of Gaussian and robust Gaussian filters for a flatness evaluation process (and measurement noise on focus variation microscopes [34]) of ground detail was taken into account. Moreover, the noise type was detected by analyzing autocorrelation coefficients for different noise [35]. The effect of some noise errors was defined and reduced [36] with various methods—for example, correlogram correlation [37,38], Fourier reduction, or random phase exclusion methods [39], by detecting limits of the roughness tester [40], limitation and matching of bandwidth for stylus or optical instruments, or the low-noise interference microscope approach [41], reproducing measurement images with Instrument Transfer Functions (ITFs) or Optical Transfer Functions (OTFs) [42], some optimization methods [43] for Coherence Scanning Interferometry measurements, z-axis repeatability studies [44], an orthogonal wavelet de-noising algorithm [45], thresholding function [46], or a comprehensively improved algorithm, which combines wavelet packet decomposition and improved complete ensemble empirical modal decomposition of adaptive noise [47].

Summarizing, the interference signal is composed of the background signal characterized by low frequency, a noise signal of high-frequency, and finally by the useful signal, which needs to be processed separately. High-frequency noise can be caused by the electrical noise in the sensor output, irrespective that nowadays, the use of sensors is more robust to environmental issues [48]. Reduction of this type of errors can provide a better resolution, which also reduces the bandwidth of the sensor. In White Light Interference (WLI), according to the non-uniform distribution of the light intensity, the extracted signal is liable to be skewed and asymmetric and to contain a lot of high-frequency noise. In general, noise increases with bandwidth. Noise is due to random fluctuations, which can contribute to a significant high-frequency component [49] of the total signal. Reduction of the bandwidth can reduce the selected amount of high-frequency noise [50]. Nevertheless, one of the main scopes of this paper is to reduce the high-frequency noise with no bandwidth modifications. When applying a wavelet transform, different frequency components of the interference signal can be essentially separated into a non-overlapping band, which is an advantage in the signal noise separation and the signal denoising process [51]. The results of surface measurement were often denoised by wavelet approaches, e.g., with an on line de-noising procedure for discrete Scanning Probe Microscopy [52], where rapid decomposition is one of the considerable benefits. Noise can be also reduced using the lifting wavelet by setting wavelet difference coefficients, e.g., containing the measurement noise with the correct oriented frequency [53]. Moreover, in the phase evolution algorithm, wavelet transform combined with soft threshold filtering and homogenization can eliminate the distortion of the interference signal [54]. With all of the above examples, wavelet transform is commonly known in S-filtration of the results of surface topography measurements. However, there is still no appropriate response to specify the influence of high-frequency measurement noise on the surface topography parameter calculation. Therefore, the effect of the improper application of data processing (noise separation) techniques and its influence on the values of surface topography parameters should also be thoroughly scrutinized.

## 2. Materials and Methods

### 2.1. Measuring Equipment and Analyzed Surfaces

**Figure 1:**
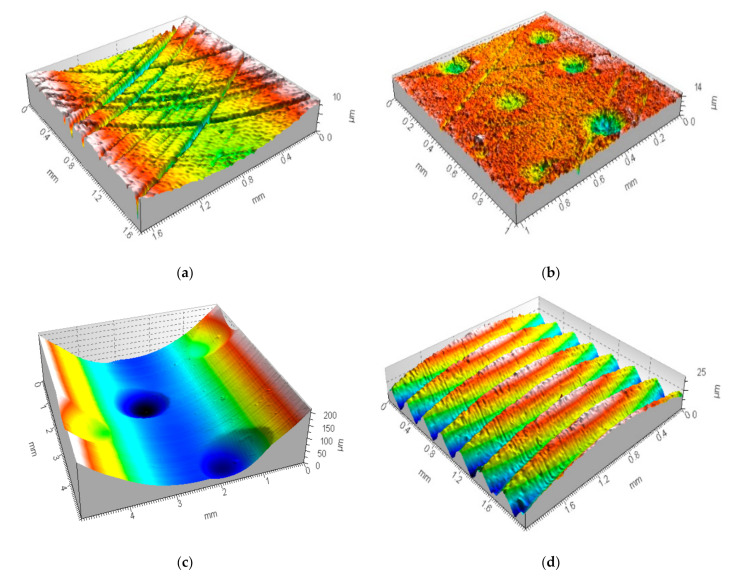

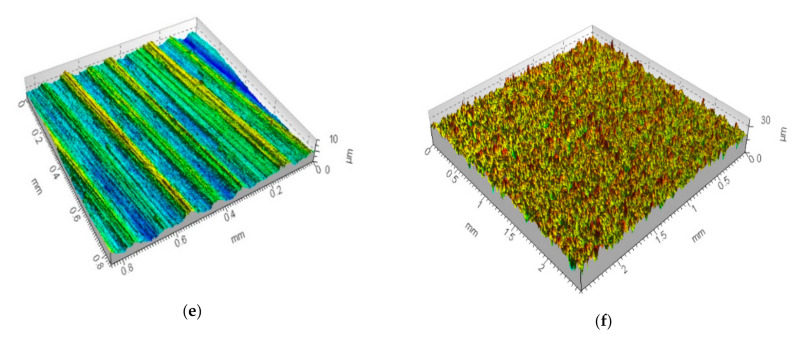
Examples of analyzed surface textures: cylinder liner texture after honing process (**a**), plateau-honed cylinder liner with additionally burnished valleys (**b**), and containing wide and deep dimples (**c**), turned piston skirt (**d**), ground (**e**) and isotropic (**f**) textures.

The following types of surfaces were taken into consideration: two-process plateau-honed cylinder liners, plateau-honed cylinder liners with additionally burnished dimples, turned piston skirts, ground and isotropic textures. Some examples of studied details are presented in Figure 1. In some cases, textures were modeled [50]. Cylindrical plateau-honed liners with burnished dimples with depth (*D*_de_) and diameter/width (*D*_di_) between 0.07 and 0.12 mm and between 0.1 and 0.8 mm correspondingly were mandatorily analyzed with the free-of-dimple specification. Free-of-dimple analysis, firstly proposed in this paper, indicates the areas of surface where oil reservoirs (oil pockets or dimples) did not occur. This type of analyzed detail is flat in general, waviness and form were eliminated; many papers contain relevant information about areal form removal of “engineered surfaces” [55,56,57,58,59,60,61] and its influence on the tribological performance, e.g., friction [62]. Therefore, the selection of roughness separation (F-operator) method was not scrutinized in the presented results. The effect of noise occurrence on areal form removal was not analyzed in this paper as well.

For areal surface topography parameter assessments, the commonly used algorithms (available in the measuring equipment software) were proposed, e.g., cylinder fitting approach based on the least square algorithm, polynomials of 2nd or 4th degrees, Gaussian regression, or robust Gaussian regression filters. When a surface texture contained deep or wide dimples, the valley-excluding method presented in previous papers of authors was also applied. To facilitate the understanding of the results obtained, more than 20 measured and 20 modeled (with generated–added dimples) surfaces were studied, but only a few of them are shown in detail.

**Figure 2:**
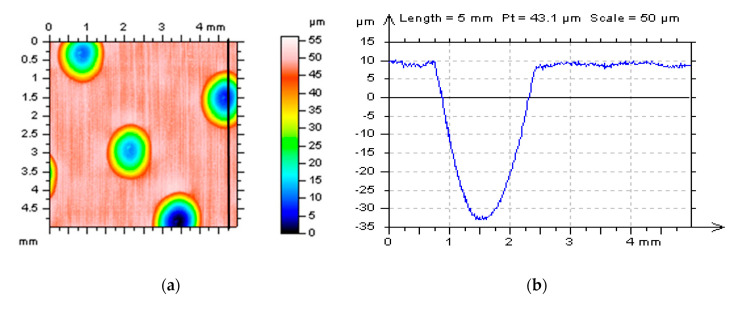

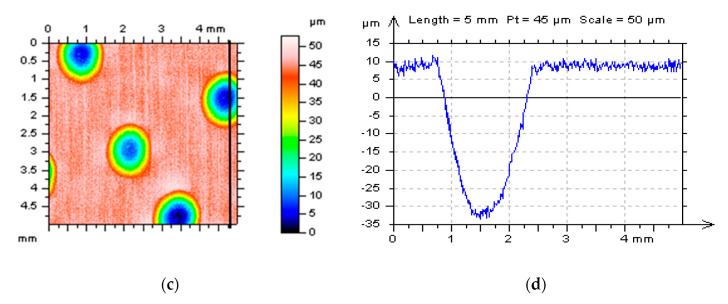
Plateau-honed cylinder liner surface: contour map plots (**a**,**c**) and profiles (**b**,**d**) containing deep (wide) oil reservoirs after different measuring speeds (MS-2 and MS-6 respectively) for stylus instrument, areas of profiles extractions were indicated on the map plots.

Considered details were measured by stylus instrument Talyscan 150 with a nominal tip radius of about 2 μm, height resolution of about 10 nm, and measurement speed (MS) from 0.1 mm/s for MS-1 to 1 mm/s for MS-10. The measurement was also provided with white light interferometer Talysurf CCI Lite with a height resolution of 0.01 nm. The measured area was 5 mm by 5 mm with 1000 × 1000 points for the stylus method or 3.35 mm by 3.35 mm with 1024 × 1024 points for the optical scheme. The sampling interval and spacing were 5 and 3.27 µm, respectively. The effect of sampling on areal texture parameters was studied in [63] and therefore was not included in the scope of the current paper. In Figure 2, cylinder liner surface topographies or profiles were presented after measurement with different conditions (MS).

The effect of errors in the detection and then reduction of high-frequency measurement noise on values of surface topography parameter was studied. The following parameters (from ISO 25178-2 standard [33]) were measured: arithmetic mean height *Sa*, auto-correlation length *Sal*, mean dale area *Sda*, root mean square gradient *Sdq*, developed interfacial areal ratio *Sdr*, mean dale volume *Sdv*, core roughness depth *Sk*, kurtosis *Sku*, inverse areal material ratio *Smc*, areal material ratio *Smr*, maximum peak height *Sp*, arithmetic mean peak curvature *Spc*, peak density *Spd*, reduced summit height *Spk*, root mean square height *Sq*, skewness *Ssk*, texture direction *Std*, texture parameter *Str*, maximum valley depth *Sv*, reduced valley depth *Svk*, extreme peak height *Sxp,* or the maximum height of surface *Sz*.

### 2.2. Proposed Methods for the Definition of High-Frequency Measurement Noise

Characterization of two process plateau-honed cylinder liner textures containing dimples was performed with the *D*_de_, *D*_di_ analysis, and—which was fairly advantageous especially when noise amplitude was relatively small—free-of-dimple details specification. Moreover, the “noise surface” (NS) was defined as “removed results” obtained by the application of the de-noising process—in particular, S-filtering approaches defined by the ISO standards [33]. Furthermore, two process textures with oil reservoirs were also considered with dimple-to-dimple (*D*_ds_) or dimple-to-edge (*D*_dte_) distances. All the above distances were received by extraction of the appropriate profiles. The accuracy of the distance determination was not taken into account.

For minimization of the influence of the noise signal, especially with high-frequency interval, the WNEP was proposed. Various type of wavelets were studied, but only a few of them, e.g., Daubechies wavelet filter of n-th order (*W*_Dbn_), Coiflet wavelet filter (*W*_Cf_), and reverse biorthogonal wavelet filter (*W*_RB_), were presented in the proposed approach. Both the Daubechies and Coiflet wavelet families are the most common orthogonal wavelets [64] and were described widely by the mathematical formulas in previous papers [65]. The Daubechies wavelet was proposed to have scaling functions with vanishing moments [66] (values), while biorthogonal wavelets are not based on the vanishing moment, and all wavelets referred to its family have a symmetric structure. Reverse biorthogonal wavelet families are guided by biorthogonal spline wavelets [67]; therefore, the symmetrical condition and reconstruction can be confirmed. The properties and applications of proposed wavelet functions were analyzed in many previous research papers [68,69]. Three proposed wavelet schemes were compared with commonly available procedures, i.e., moving average (MAF), median (MF), and Gaussian (GF) filters. Initially, the detection of high-frequency noise presence was suggested with a PSD or autocorrelation function (ACF) appliance.

The accuracy of the high-frequency noise reduction procedure was also defined with parameter coefficient (*P*_Coef_) or parameter difference coefficient (*P*_DiffCoef_) calculation and the NS analysis. Both coefficients were expressed in percentage (%) and were proceeded for modeled high-frequency errors. The *P*_Coef_ or *P*_DiffCoef_ were calculated as the sum of the relative differences of parameters defined as the most susceptible to the influence of high-frequency errors. From four parameters, each was selected from various parameter groups: height, hybrid, feature, and functional. Both the *P*_Coef_ and *P*_DiffCoef_ coefficients were calculated as a relative difference calculated for parameters before adding a high-frequency noise and after its removal. For each parameter, we assigned weight values equal to 0.25. The effect of various types of weight was not considered. The WNEP is based on the minimization of values of these two (*P*_Coef_ and *P*_DiffCoef_) coefficients.

## 3. Results and Discussion

Characterization of high-frequency measurement noise was divided into three parts. In the first Section 3.1, the influence of some features of surface texture (e.g., valley, dimples, oil pockets) on the detection process of noise was presented. The influence of dimple size on noise recognition was not considered in this paper because it was placed in the previous research [70]. Except for the eye-view characterization of measurement noise, the PSD and ACF were applied for noise definition. Moreover, the effect of high-frequency measurement noise on the values of surface topography parameters of plateau-honed cylinder liner textures was presented. It was found that the characterization of measurement noise with PSDs or ACFs might not give quantitative information when some surface topography features are found in the analyzed detail (profile). Therefore, the problems in noise definition were accurately identified in Section 3.2. Moreover, the reduction of data processing (noise removal) errors with its effect on values of the surface topography parameters (from ISO 25178 standard) was studied in Section 3.3.

### 3.1. The Influence of Measurement Noise on the Values of Surface Texture Parameters with Selected Features (Valleys) Analysis

**Figure 3:**
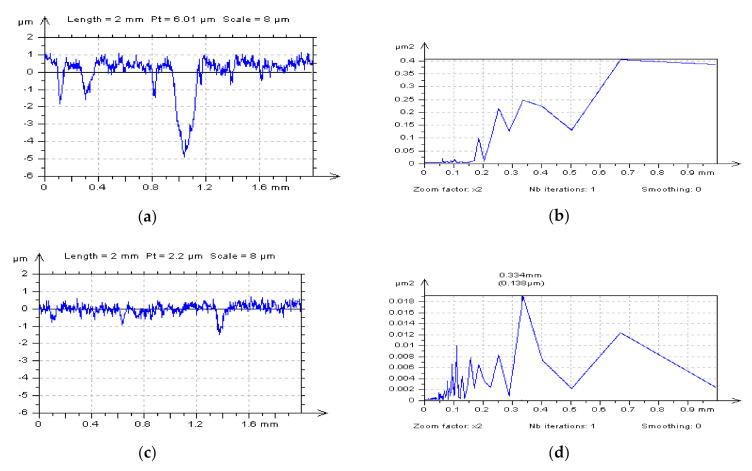
Profiles extracted from the plateau-honed texture (after form separation by application of a polynomial of second degree): containing oil reservoir (**a**), free-of-dimple detail (**c**), and their power spectral densities (PSDs) (**b**,**d**) correspondingly.

Noise, especially noise amplitude, can straightly be defined by calculating the difference from two measured details, results of two type of measuring by the same method, in particular. In fact, NS can be proposed by subtracting two measured results. However, there is still a marvelous problem for defining the “noise results” of measurement, especially when the non-contact method is applied. When plateau-honed cylinder liner surface textures with burnished oil pockets were considered, it was assumed that the characterization of PSD did not allow detecting the high-frequency noise from the results of surface topography measurements. When free-of-dimple profiles were considered, the high-frequency noise presence was instantly recognizable (Figure 3). Assessment of profiles that included dimples for high-frequency noise occurrence was not unequivocal. Therefore, it can be suggested to detect the high-frequency noise with PSD of free-of-dimple profiles (or areas) analysis. For a plateau-honed cylinder liner surface (or for plateau-honed cylinder liners with dimples created by the burnishing techniques), the high-frequency noise was (in some cases) extracted with some features, e.g., scratches or dimples. Additionally, when dimples were located on the edge of the analyzed detail, the high-frequency noise was removed with other features. Consequently, it is recommended to suppress the high-frequency noise with studies of the isometric view of the surface.

**Figure 4:**
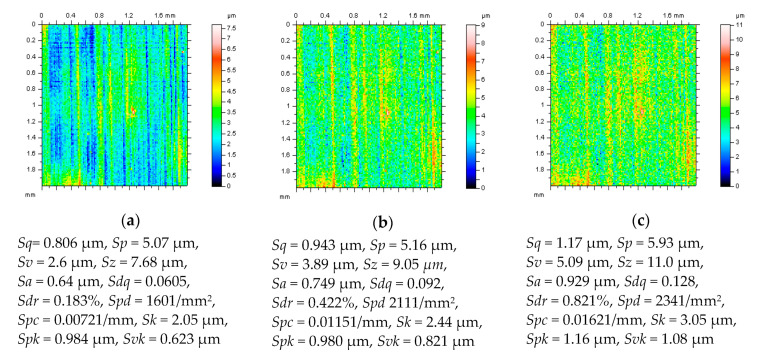
Free-of-dimple details (and their parameters correspondingly) from a cylinder liner surface after various stylus measuring conditions, MS-2 (**a**), MS-4 (**b**) and MS-6 (**c**) and application of the least-square fitted cylinder plane approach with the valley excluding approach.

When free-of-dimple details from a cylinder liner surface containing deep and (or) wide valleys were taken into consideration (Figure 4), it was assumed that an increase of high-frequency noise amplitude caused significant differences for the values of the *Sk*, *Spk,* and *Svk* parameters (after processing by the least-square fitted plane of a polynomial of 2nd or 4th degrees with valley excluding approach), and only small (usually negligible) differences in PSDs were found. Moreover, the *Sdq*, *Sdr*, and *Spd* parameters increased the most, height parameters also increased between 10% and 30%, while the value of *Sal* decreased (in some cases) by more than 50%. When the amplitude of high-frequency noise grew, the *Sq*, *Sp*, *Sv*, *Sz,* and *Sa* parameters also grew by 0.85%, 14.13%, 10.06%, 12.08%, and 1.27%, respectively (for the noise with the most significant amplitude). However, some of the height parameters were decreased such as *Ssk* and *Sku* between 0.45% and 2.25% and between 0.28% and 1.70%, respectively. The *Smr* parameter decreased between 43.48% and 96.66%, but *Smc* increased between 1.03% and 16.75%. The *Sxp* value had no change due to the high-frequency noise presence. The spatial parameters (*Sal*, *Str,* and *Std*) had no modification except for a barely noticeable difference (usually less than 0.2%). Both hybrid and feature parameters undergo significant variations: the *Sdq* (*Sdr*) parameter grew between 41.90% (112.95%) and 414.29% (2672.02%); *Spd* (*Spc*) increased 8108.33% (4066.67%), and *Sda* decreased 93.24%, especially when the amplitude of high-frequency noise increased more than twice. For high-frequency noise presence, the most sensitive are *Smr*, *Sdq*, *Sdr*, *Spd,* and *Spc* parameters (Table 1). Moreover, in some cases (for selected types of surface textures), the values of the texture parameters can arise more than 100% (especially when confocal measurement techniques are applied), e.g., for the *Sda* parameter, then the proposal of minimization (reduction) of the results of high-frequency noise presented can be crucial for the elimination of data processing errors in surface quality assessments.

It was also found, for all of the types of analyzed surface topographies, that areal surface topography parameters (except *Sda* and *Sdv*) changed proportionally due to growth of the high-frequency noise amplitude, which increased or decreased along with all the high-frequency noise occurrence. This proportionality might be tremendously useful to define (select) the procedure for the detection of high-frequency noise from all types of surfaces according to PSD included (calculated) in commercial software.

**Figure 5:**
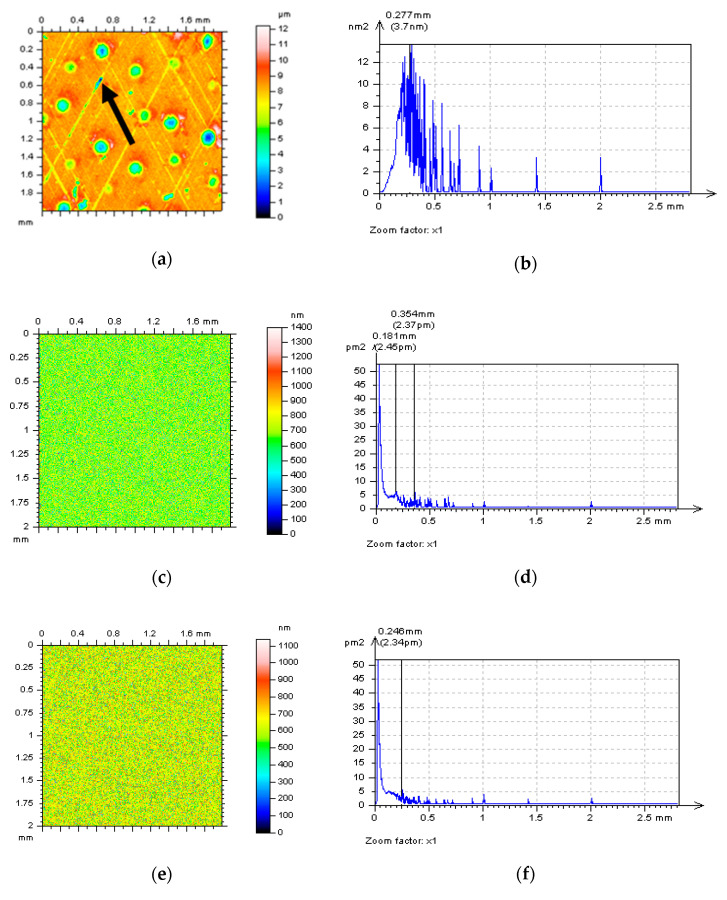

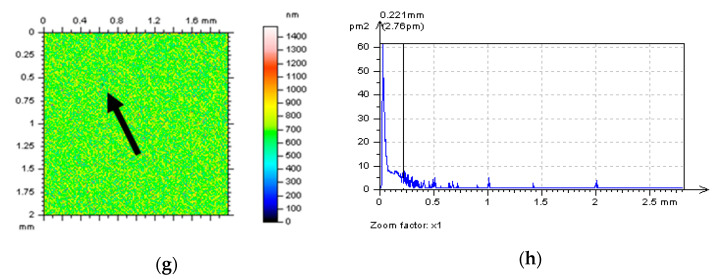
Isometric views measured by the Scanning White-Light Interferometry (SWLI) method: (**a**) plateau-honed cylinder liner surface with extraction of the high-frequency noise by *W*_Db4_ (**c**), *W*_Cf_ (**e**), or *W*_RB_ (**g**) and respectively their PSDs (**b**,**d**,**f**,**h**).

For reduction of the influence of high-frequency noise on the results of surface topography measurement, various algorithms (*W*_Dbn_, *W*_Cf_, and *W*_RB_) were proposed. In this case, removed from the raw measured data, the high-frequency components were defined as an NS. False estimation of high-frequency noise (NS) was recognized when valleys (scratches) with sharp edges were found (Figure 5a). In the previously presented Figure 5g, a small-scale area of unexpected NS deformation was indicated by the arrow. When PSD was taken into account, all the proposed procedures gave a correct and acceptable solution. However, some deformations of NS were observed. Therefore, analysis of the isometric view of studied details was suggested with the application of data processing methods, e.g., WNEP.

**Figure 6:**
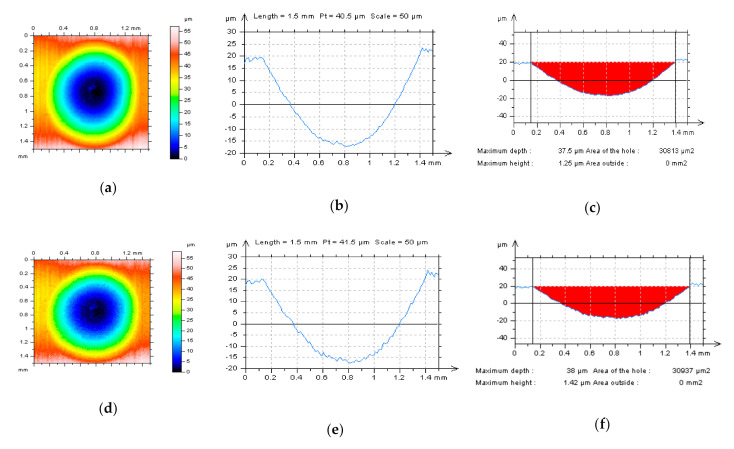

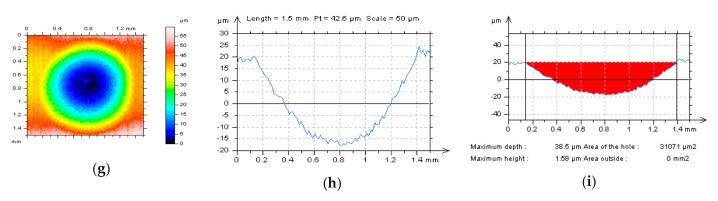
Isometric views (**a**,**d**,**g**) of detail from plateau-honed cylinder liner surface topography containing dimples (*D*_de_ = 40 µm, *D*_di_ = 1.2 mm in average approximately); example profiles (**b**,**e**,**h**) and their hole/peak area diagrams (**c**,**f**,**i**) after various measurement speeds (MS: MS-2, MS-4, and MS-6) correspondingly.

When the analyzed details contained deep (wide) valleys (example in Figure 6), it was automatically assumed that the valley maximum depth, valley maximum height, and valley area of the whole increased 3%, 26%, and 1%, respectively when MS (and NS amplitude simultaneously) increased from 0.2 to 0.6 mm/s. The valley area outside has no change regardless of the MS value. It was shown that with valley density (suitable area density, e.g., from 7.5% to 20%) and valley area of the whole (with the proper shape, e.g., spherical or short/long-drop shape dimples, and dimensions) both accurately defined, the friction characteristics of the sliding pairs could be improved in comparison to non-textured surfaces [71]. False estimation of oil-reservoir volume can cause a classification of a properly made part as a lack and its rejection. This research is all the more important because it was noticed that oil emission by the engine was proportional to the value of the *Sk* parameter, and cylinder wear under various conditions was proportional to the value of the surface emptiness coefficient *Sp*/*Sz* (SEC) [72,73].

Selection of the procedure for high-frequency noise extraction might be offered for NS created by the Wavelet applications. This NS should contain the biggest values (amplitudes) of high frequencies for noise characterization with surfaces or profiles analysis. The biggest amplitude of the high-frequency components of the removed NS, as received by the application of the WNEP, contains the better results that might be obtained from the suggested data processing method.

**Figure 7:**
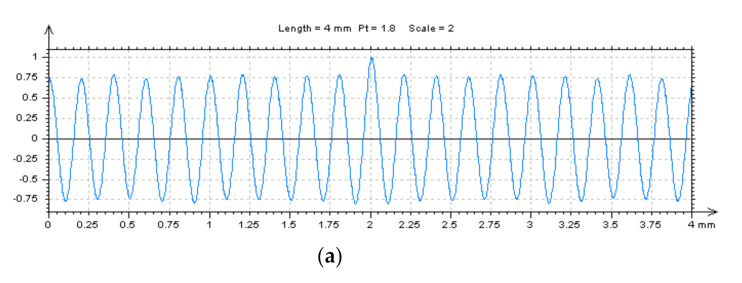

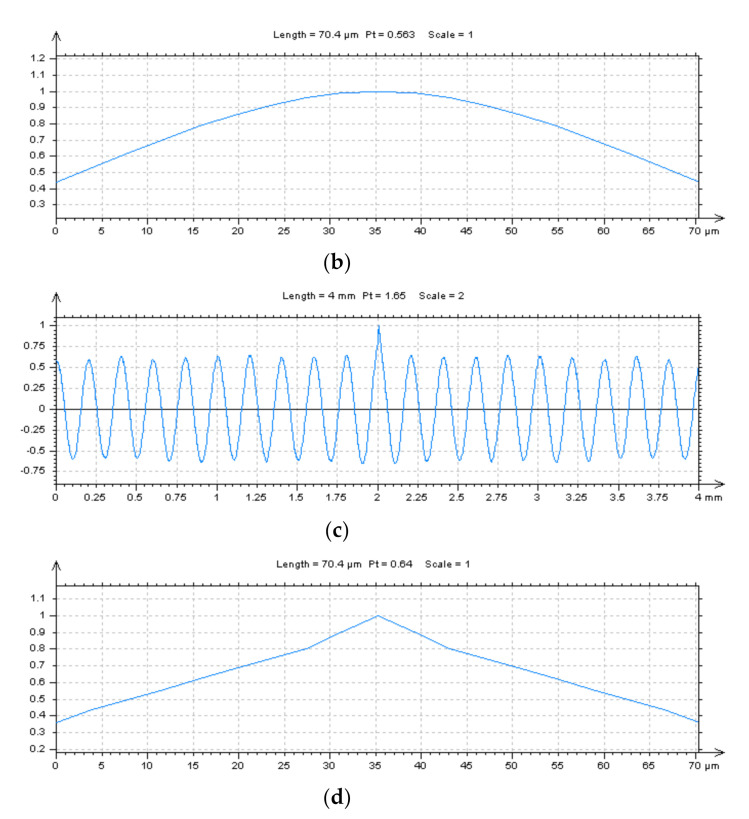
Autocorrelation functions (ACFs) (**a**,**c**) and its enlarged middle part (**b**,**d**) for the turned piston skirt surface in Table 2 (**a**,**b**) or MS-7 (**c**,**d**) conditions.

For minimization of errors in the calculation of surface texture parameters, the ACF (defined for surface) can be also applied, especially when noise detection is required. In Figure 7, the ACF for a turned piston skirt surface was presented. It was noticed that there were only small (often slight) variations when the shape of function (characteristic) was analyzed. However, when the middle part of ACF was taken into account (e.g., it was analyzed in the enlarged form), substantial changes were instantly recognized. Similar conclusions might be accomplished for ground details. When plateau-honed cylinder liner textures were studied, the “reshape modifications” of the ACF were particularly noticeable, regardless of the dimple occurrence. For additionally burnished valleys, the ACF form modification was difficult to notice. The biggest (smallest) values of *D*_de_ and *D*_di_ (*D*_ds_) were received the increasement (reduction) in ACF transformation was obtained. Therefore, as it was already mentioned, for plateau-honed cylinder liners with deep (wide) dimples, it is suggested to define the high-frequency noise (NS) occurrence with out-of-valley (dimples, oil pockets) analysis.

### 3.2. Problems in Definition and Extraction of Noise from the Results of Surface Topography Measurements

**Figure 8:**
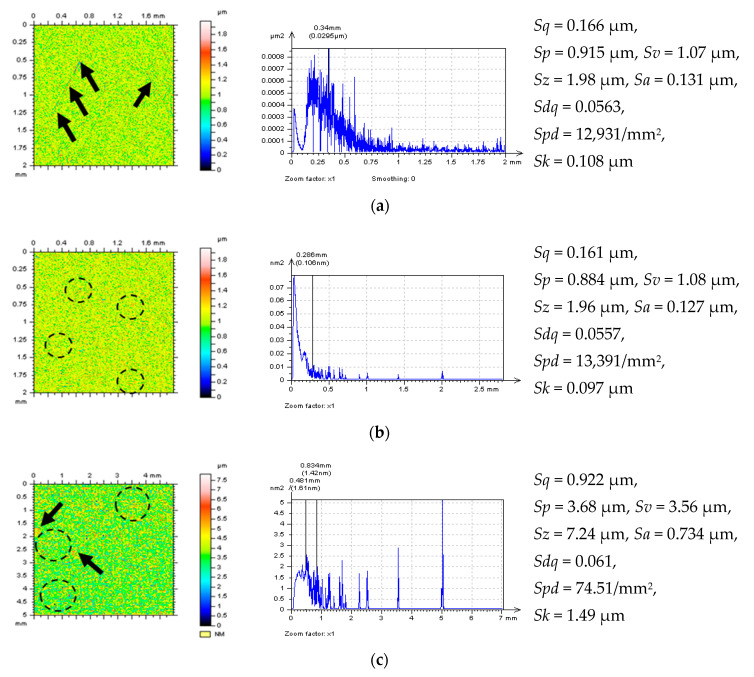
Decomposed “noise surface” (NSs) by the Wavelet Noise Extraction Procedure (WNEP) from a plateau-honed cylinder liner surface (**a**) with additionally added oil pockets (**b**), or containing deep and wide dimples (valleys) (**c**) with PSDs and selected parameters, respectively.

**Figure 9:**
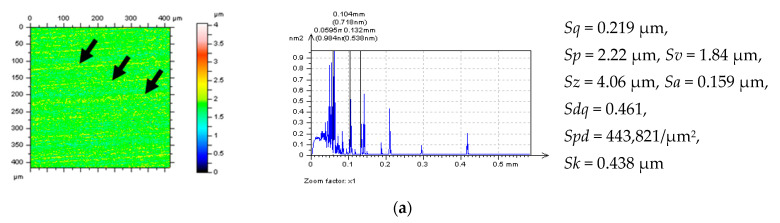

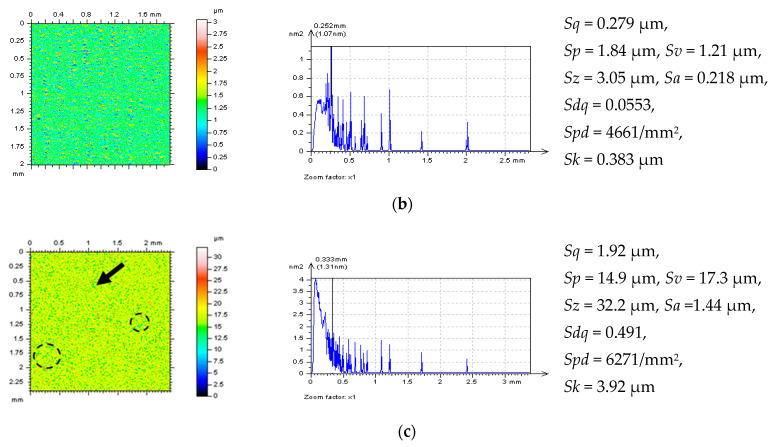
NSs (**left column**) with their PSDs (**median column**) and selected parameters (**right column**), decomposed by application of WNEP from the ground (**a**), turned (**b**) and isotropic (**c**) surface topographies.

It was noticed that for surfaces with spacing greater than 5 µm, the detection of high-frequency noise can be exceedingly difficult when analysis of the isometric view of the surface (or profile) is taken into account. It was also previously found (in the last researcher issues) that when the number (density) of dimples (features in general) was greater than 0.2 mm (*D*_ds_ < *D*_di_ for surface containing oil pockets), the detection and extraction of high-frequency noise were also increasingly complicated. It can be directly observed when both process textures and surfaces with additionally burnished dimples are taken into consideration (Figure 8). When NS is defined, some features are removed from analyzed detail as well. For ground details, the machining trace can be especially noticeable—it was indicated by the arrows in Figure 9. Some of the parts of Figure 8 and Figure 9 are presented in enlarged details.

For a plateau-honed cylinder liner surface (same as for plateau-honed cylinder liners with additionally burnished dimples), the high-frequency noise was (in some cases) decomposed with some scratches or dimples, especially when dimples were located on the edges of the considered detail (*D*_dte_ = 0). Then, the high-frequency noise was removed with other features, and selected features could be found on the received NS. Therefore, it is recommended to extract (decompose) the high-frequency noise with the studies of an isometric view of the analyzed surface. There is still an insurmountable problem for edge-effect minimization when decomposition (e.g., the process of form and waviness removal or procedure of measurement noise reduction) occurs. It was found that the smallest *D*_dte_ was calculated when the biggest distortions of surface topography parameters appeared.

For the minimization of errors of the high-frequency noise removal procedure, analysis of the standard deviation plane (SDP) can be also applied. SDP is defined as a difference calculated for the measured surface and the surface after high-frequency noise removal. When the amplitude of the SDP increased, the errors of the surface topography parameter calculation also were enlarged. The results (differences defined with the SDP) obtained after WNEP application can be compared with the average method (average results from 10 measurements). However, these proposals have not been studied in the current paper. 

When a surface detail contains features (scratches or valleys) with sharp edges, the definition of both NS and the procedure for removal of the high-frequency noise can be exceedingly difficult. The problem of analysis of surface texture edges can be considerably enlarged for both *D*_di_/*D*_de_ and *D*_ds_/*D*_dte_ consideration. Increasing the values of *D*_di_ and (or) *D*_de_ variables caused an enlargement of errors in the calculation of surface topography parameters. However, when the values of *D*_ds_ and *D*_dte_ decreased, the errors usually decrease regardless of the enlargement in the size (*D*_di_ or *D*_de_) of dimples (features). When other frequencies (details) than those in the high-frequency domain (e.g., in the Figure 8a) were found on the NS, then the procedure of reduction of a high-frequency noise should be replaced by another algorithm. For isotropic textures, some scarcely noticeable features (Figure 9b) can be conspicuously omitted when the *P*_Coef_ or *P*_DiffCoef_ value is minimized. Therefore, the analysis of the PSD graphs should be exhaustively discussed.

Usually, the highest number of unanticipated and noteworthy features was noticed when the NS was thoroughly evaluated from the isotropic textures, considering all of the types of (considered in this paper) measured surfaces. For plateau-honed cylinder liner textures, the scratches on NS were observed generally; when the surface contained dimples (valleys), the scratches (treatment traces) were virtually imperceptible. Moreover, when *D*_di_ and *D*_de_ increased (*D*_de_ > 30 µm and *D*_di_ > 0.4 mm), the treatment traces (features) disappeared entirely from the received NSs. However, the occurrence of deep and wide valleys caused the areas of a gathering of the measurement noise to also be found on the NSs. It was especially noticeable in the areas where the sharp edges, scratches, or dimples were located. What is more, the areas of noise convergence were readily recognizable when *D*_dte_ < 0.2 mm for valleys with *D*_de_ > 30 µm and *D*_di_ > 0.4 mm. Generally, when *D*_dte_ < 4 × *D**_di_***, then the ‘noise gathered’ areas could be directly perceived for two-process textures with additionally burnished oil pockets. Moreover, the number (density) of ‘noise gathering’ areas also increased when dimple-to-edge areas were studied, as indicated by arrows in Figure 8c. The characterization (analysis) of NS by the reduction (removal) of the ‘noise gathering’ areas can be valuable in the selection of procedure for minimization of the effect of the high-frequency noise on the results of surface topography measurements.

The reduction of noise influence on the surface topography parameters can be also originally proposed by the analysis of the noise (NS in particular) amplitude. The smallest (greatest) *Sz* value (maximum height) of NS was found (deliberately excluding isotropic topographies) for plateau-honed textures (with the deepest valleys), which was 7.24 µm (1.98 µm) for established details. The *Sz* value of NS from the isotropic texture was fairly bigger; notwithstanding, the values of *Sz* of the measured surface was proportionally greater as well. It was also found that the NS extracted from a turned topographies does not (usually) contain the ‘noise gathering’ areas. Nonetheless, the treatment traces are much more particularly noticeable than for dimple-containing plateau-honed textures, irrespective of values of the size of the dimple (*D*_di_ and *D*_de_).

Some unnecessary (essential for surface texture parameter calculation of a surface with processed raw measured data) features of NS can be visible with PSD consideration; the occurrence of high-frequency noise for plateau-honed textures (when the surface did not contain oil reservoirs or *D*_di_ and *D*_de_ is relatively small, which has already been adequately described previously) can be likewise precisely observed (Figure 8a,b). The scrutiny of PSD can be completely unsuitable for the detection and reduction of the high-frequency noise in turned or ground topographies measurements.

### 3.3. Reduction of Errors in the Noise Removal Process

To minimize the effect of noise detection and reduction, a procedure (algorithm) is being suggested. Therefore, the modeled textures were carefully analyzed. For flat surfaces (after the form removal process), the high-frequency noise was added and then removed by various filters. In the previous results (in the past papers), it was assumed that the values of *Sz*, *Sdq*, *Spd*, and *Sk* parameters were changed the most, and simultaneously, the degree of change in value was proportional to the amplitude of the measurement errors when a high-frequency noise was indicated in the received measurement data. Consequently, those four parameters were defined as (the most) ‘noise-sensitive parameters’ (NSP) as it follows. However, the number of NSP can be modified when various types of surface texture are considered. In general, the above four parameters are sensitive for high-frequency noise occurrence for each type of surface topography analyzed in this paper. The *P*_Coef_ or *P*_DiffCoef_ were proposed by the sum of the relative differences of those parameters (each from various parameter groups: height, hybrid, feature, and functional). The *P*_Coef_ (or *P*_Di*f*fCoef_ respectively) were calculated as a relative difference calculated for parameters before adding a high-frequency noise and after its removal. For each parameter, a weight value equal to 0.25 was assigned. The effect of various types of weight was not considered in this paper; nevertheless, the comprehensive studies on the value of this factor should be determined in future research. Values of *P*_Coef_ and *P*_DiffCoef_ were expressed as a percentage, but to simplify the analysis, the unit has not been reported in some (most) cases.

**Figure 10:**
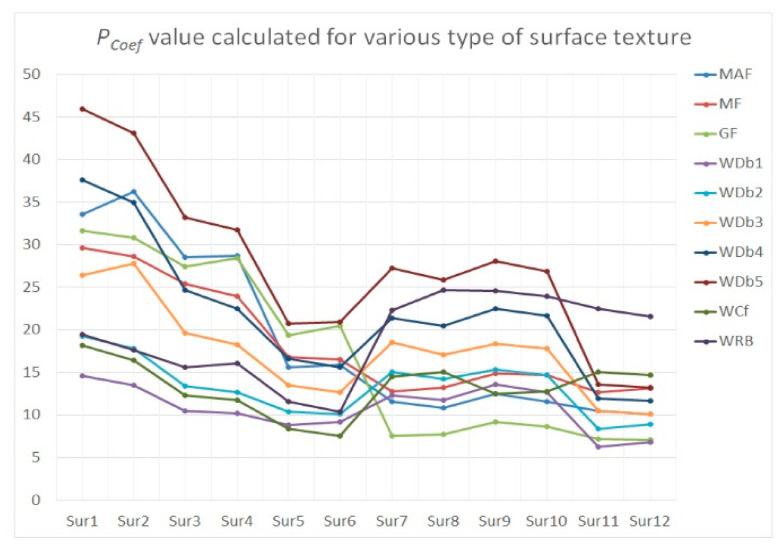
The *P*_Coef_ values calculated for the minimization of high-frequency noise errors.

**Figure 11:**
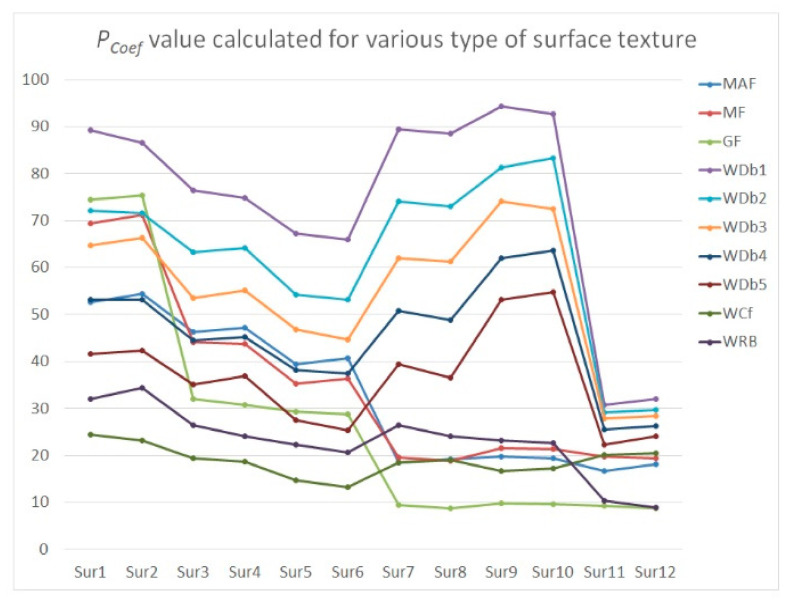
The *P*_Coef_ values described for surfaces measured with noise amplitude approximately equal to the average value of MS-6, MS-7, MS-8, MS-9, and MS-10 noise amplitude.

When the values of *P*_Coef_ were found, the six algorithms (three commonly used and available in measurement equipment software, e.g., filters: MAF, MF, or GF, and newly proposed *W*_Dbn_, *W*_Cf_, and *W*_RB_ approach) were applied and compared for reduction of the effect of high-frequency measurement noise occurrence. The analysis was proposed for each type of surface as follows: plateau-honed cylinder liner (Sur1, Sur2) with additionally burnished valleys (Sur3, Sur4) or with deep and wide dimples (Sur5, Sur6), turned piston skirts (Sur7, Sur8), ground (Sur9, Sur10) and isotropic (Sur11, Sur12) textures. The amplitude (*Sz*) of the added NS component was approximately similar to the amplitude (*Sz*) of NS defined for measurements from MS-1 to MS-5 (Table 2 and Figure 10) and from MS-6 to MS-10 (Figure 11), respectively.

When the different amplitudes of high-frequency noise were applied, some procedures gave non-lasting and unrepeatable results. For plateau-honed cylinder liner surface textures, the application of *W*_Db1_, *W*_Cf_, and *W*_RB_ caused a smaller distortion of NSP than commonly used procedures. The distortion increased when the degree of *W*_Dbn_ also increased. Notwithstanding, the higher the NS amplitude that appeared, the smaller the NSP exaggeration that occurred. Increasing the *W*_Dbn_ degree is suggested for surfaces measured with higher (depending on the surface texture type) MS values. When the distortion of the raw measured data for a cylinder liner surface (contained additionally burnished dimples and (or) scratches) decreased (for all of the applied filters in practice), the smallest value of *P*_Coef_ was achieved when the *W*_Cf_ was applied. When *D*_de_ and *D*_di_ increased, the *P*_Coef_ value decreased, which can be caused by a smaller distortion of parameters when deep and wide valleys occurred (this was mentioned in the first section when PSD was proposed for high-frequency noise occurrence detection with free-of-dimple details specification).

For turned or ground details, it was assumed that the lowest values of *P*_Coef_ were remarked with Gaussian filtering regardless of the value of the amplitude of high-frequency noise; hence, the *P*_Coef_ value increased when the NS amplitude increased slightly according to the other applied algorithms. From (three) proposed wavelets, the *W*_Db1_ (*W*_Cf_) lead to the particularly valuable and direct results for turned (ground) details. The value of *P*_Coef_ increased (decreased) when the degree of *W*_Dbn_ was enlarged (reduced) for MS ≥ MS-6 (MS ≤ MS-5). 

Application of the WNEP scheme (approach for the minimization of influence of measurement noise) for isotropic surfaces led to desired results when *W*_Db1_, *W*_Db2_, or GF (in particular instances) were accomplished. The usage of *W*_RB_ caused an enlargement of *P*_Coef_ value by more than 200% by the *W*_Db1_ or Gaussian filtering irrespective of MS (MS ≤ 5 or MS > 5). On average, application of the *W*_Dbn_ (from *W*_Db1_ to *W*_Db5_ degree) filtering method caused the minimization of the *P*_Coef_ value for isotropic textures under all (six) studied filtering methods.

Another approach can be provided by direct counting the differences of surface topography parameter(s) between surfaces measured with different velocities, e.g., MS-1 and MS-10. When smaller differences between MS-1 and S-filtered MS-10 surface texture parameters (identified as an NSP) were obtained, the superior influence of the proposed algorithms on the minimization of surface topography parameter distortions was perceived. To confirm the received results, the measurement (with MS-1 or MS-10) was repeated 10 times; then, the average value of measurement results was taken into detailed consideration.

**Figure 12:**
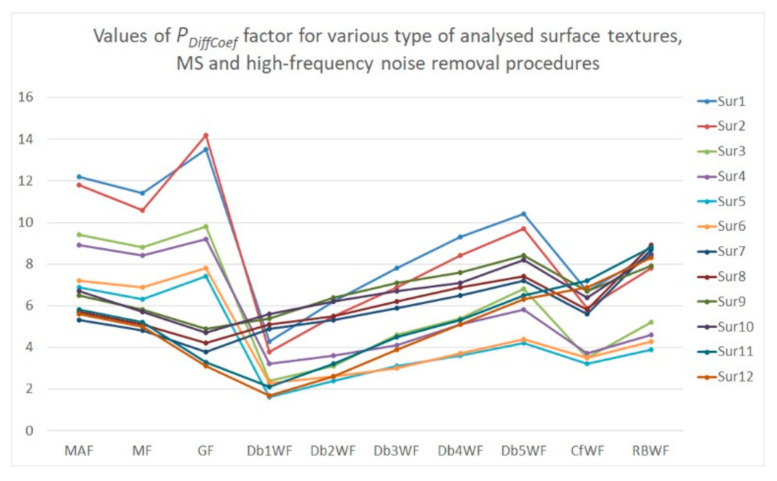
The value of *P*_DiffCoef_ calculated for the difference between MS-1 measured surface and MS-10 measured surface with noise removed by various data processing methods.

It was found that the errors of values of surface topography parameters after the application of commonly used data processing (noise removal) methods (e.g., MAF, MF, or GF) decreased when the *D*_de_ and (or) *D*_di_ increased. Accordingly, the effect of NS on surface topography parameters increased when the dimples size decreased. Application of the *W*_Db1_ filtering method caused the minimization of the value of *P*_DiffCoef_ (description in Figure 12) when plateau-honed cylinder liner textures were considered. When the biggest degree of *W_Dbn_* was applied, the biggest distortions of the surface texture parameter were found. For turned or ground (isotropic) details, the GF (*W*_Db1_) provided the desired results—the minimum value of *P*_Coef_ was obtained. Thus, from generally used (proposed in commercial software) methods, the Gaussian filter (MF for plateau-honed cylinder liner surfaces) might give the most effective solution for minimization of the noise in surface texture measurements. A properly defined NS should contain only the high frequencies, specifying frequencies in the required (considered) domain, when PSD is taken into account. Therefore, in the control process, the NSs (especially its PSDs) should be also systematically analyzed. Moreover, ‘unexpected’ features on the NS (e.g., scratches or edges of selected features) should be found infrequently. When procedures for the separation of NS from the results of surface texture measurement are selected arbitrarily (randomly), then some non-high-frequencies are readily visible in both isometric views and PSD graphs. In some cases, the analysis of the isometric view of surface (and PSD graph) as well as minimization of the *P*_Coef_ and *P*_DiffCoef_ coefficients may not be entirely convincing. Consequently, the multivariate analysis (minimization of the *P*_Coef_ and *P*_DiffCoef_ coefficients, assessment of the NS, PSD, and ACF) may be reasonably required, especially when various types of surface topographies are studied.

## 4. Conclusions

It is particularly complicated to define the measurement noise, especially in the high-frequency domain, to reduce the effect of raw measured data processing (S-filtering) errors. Despite that, some conclusions can be drawn:

- When high-frequency noise is noticed in the results of surface topography measurements, the values of the surface texture parameters can be erroneously determined. Some of them were overestimated by more than 100%. Those types of parameters might be specified as ‘noise-sensitive parameters’ (NSP), and their detailed analysis might be especially relevant in the process of minimization of the influence of noise occurrence on the values of the surface topography parameters.
- High-frequency noise can be characterized by the analysis of the ‘noise surface’ (NS) as a result of the application of the noise removal (reduction) algorithm, e.g., filtering. Properly defined (received by the application of properly selected filtration algorithm) NS should contain only those irrelevant (in the required measured data) components of the analyzed surface data. When NS contained components with other frequencies (other than high frequencies), e.g., scratches or valleys, then the proposed filtration algorithm should not be considered for the characterization (detection and reduction) of the surface topography measurement noise.
- Selection of the procedure for measurement noise reduction might be affected by the number (density), distance (dimple-to-dimple *D*_ds_ or dimple-to-edge *D*_dte_), and the size (depth *D*_de_ and diameter/width *D*_di_) of the features from surface texture, e.g., scratches, valleys, dimples, or oil pockets. The effect of values of *D*_ds_, *D*_dte_, *D*_de_, and *D*_di_ on the process of both the detection and reduction of the high-frequency measurement noise was studied.
- As originally proposed in this paper, the Wavelet Noise Extraction Procedure (WNEP), which is based on the minimization of differences of NSP, can be valuable in reduction of the high-frequency measurement noise. In this research, three of the wavelets were compared with regularly used filters, but this minimization approach can be applied for various types of filtering methods.
- It is suggested to select the filtering method according to the type of analyzed texture as well, so for the plateau-honed cylinder liner topographies that additionally contain oil pockets, the Coiflet wavelet might have given encouraging results, out of all of the analyzed filtering methods, in the suppression of the high-frequency measurement noise. The Daubechies wavelet of 1st degree can be applied alternatively. When turned or ground surfaces are analyzed, the regular Gaussian filter can provide a marked effect in the reduction of the noise. When the isotropic details are studied, the Daubechies wavelet can be certainly applied.
- For all of the types of analyzed details, it was noticed that values of areal surface texture parameters (excluding mean dale area *Sda* and mean dale volume *Sdv*) changed proportionally due to enlargement of the high-frequency noise amplitude. This proportionality might be highly advantageous for the selection of procedure for high-frequency noise detection from all types of surfaces. Consequently, the analysis of PSD function (included in commercial software) can be a direct confirmation of the sentence above.
- Generally, to provide more precise detection and reduction (minimization) methods of the high-frequency measurement noise, the multivariate analysis might be necessary. Thus, the minimization of the parameter (*P*_Coef_) and the parameter difference (*P*_DiffCoef_) coefficients with simultaneous analysis of the NS, PSD, and ACF might be reasonably required.

In the future, the results of detection and reduction of the high-frequency measurement noise from the raw measured data of milled, laser-textured, composite, or ceramic topographies will be published by the author.

## Figures and Tables

**Table 1:** Surface topography parameters of detail extracted from the plateau-honed cylinder liner surface measured by the stylus method with different speed (from 0.1 to 1 mm/s).

Parameters of Surface Measured with Different Stylus Conditions (Speed)										
Method	MS-1	MS-2	MS-3	MS-4	MS-5	MS-6	MS-7	MS-8	MS-9	MS-10
*Sq*	23.6	23.6	23.7	23.7	23.7	23.8	23.8	23.8	23.9	24.0
*Ssk*	−2.22	−2.22	−2.21	−2.20	−2.19	−2.17	−2.17	−2.16	−2.14	−2.13
*Sku*	7.04	7.03	7.02	6.99	6.96	6.92	6.90	6.88	6.85	6.83
*Sp*	53.8	54.2	55.0	56.1	58.9	61.4	62.1	63.2	64.4	66.1
*Sv*	95.4	96.7	97.4	100.0	101.0	105.0	107.4	109.1	110.8	111.7
*Sz*	149.2	150.9	152.4	156.1	159.9	166.4	169.5	172.3	173.2	177.8
*Sa*	15.7	15.8	15.8	15.8	15.8	15.9	15.9	15.9	16.0	16.0
*Smr*	0.00299	0.00169	0.00030	0.00037	0.00009	0.00009	0.00009	0.00009	0.00008	0.00007
*Smc*	38.8	39.2	39.8	40.7	43.2	45.3	47.2	48.8	49.6	50.3
*Sxp*	83.0	83.0	83.0	82.9	83.0	83.0	82.9	83.0	83.0	83.0
*Sal*	0.434	0.434	0.434	0.434	0.434	0.434	0.434	0.434	0.434	0.434
*Str*	0.842	0.842	0.842	0.843	0.843	0.843	0.843	0.843	0.843	0.843
*Std*	90.0	89.9	89.9	90.0	90.0	90.0	90.0	89.9	90.0	90.0
*Sdq*	0.210	0.298	0.472	0.667	0.870	1.080	1.167	1.283	0.421	1.550
*Sdr*	1.93	4.11	10.6	21.1	35.4	53.5	75.4	104.1	146.4	198.5
*Spd*	0.240	0.400	0.480	0.799	4.560	19.700	34.560	79.700	104.560	196.700
*Spc*	0.0156	0.102	0.209	0.390	0.548	0.650	0.745	0.860	0.948	1.150
*Sda*	6.730	8.280	6.690	4.780	0.978	0.455	0.354	0.223	0.198	0.175
*Sdv*	0.0185	0.0219	0.0168	0.0125	0.00435	0.00017	0.00012	0.00011	0.00008	0.00007

**Table 2:** Values of P_Coef_ for various type of surface textures and high-frequency noise removal (reduction) algorithms.

Measured Surface with Noise Amplitude Approximately Equal to the Average Value of MS-5 Noise Amplitude												
Method	Sur1	Sur2	Sur3	Sur4	Sur5	Sur6	Sur7	Sur8	Sur9	Sur10	Sur11	Sur12
MAF	33.6	36.2	28.5	28.7	15.6	15.9	11.6	10.8	12.5	11.6	10.5	10.1
MF	29.6	28.6	25.4	23.9	16.8	16.5	12.8	13.2	14.9	14.7	12.7	13.1
GF	31.6	30.8	27.4	28.4	19.4	20.5	7.5	7.7	9.2	8.6	7.2	7.1
*W*_Db1_	14.6	13.5	10.5	10.2	8.8	9.2	12.3	11.8	13.6	12.7	6.3	6.8
*W*_Db2_	19.3	17.8	13.4	12.7	10.4	10.1	15.1	14.2	15.3	14.7	8.4	8.9
*W*_Db3_	26.4	27.8	19.6	18.3	13.5	12.7	18.5	17.1	18.4	17.8	10.5	10.1
*W*_Db4_	37.6	34.9	24.7	22.5	16.6	15.6	21.4	20.5	22.5	21.7	11.9	11.7
*W*_Db5_	45.9	43.1	33.2	31.7	20.7	20.9	27.2	25.9	28.1	26.9	13.6	13.2
*W*_Cf_	18.2	16.4	12.3	11.8	8.4	7.5	14.5	15.1	12.5	12.8	15.1	14.7
*W*_RB_	19.5	17.6	15.6	16.1	11.6	10.4	22.3	24.7	24.6	23.9	22.5	21.6

## Data Availability

Data sharing is not applicable to this article.

## Parameters and Abbreviations

The following abbreviations (left column) and parameters (right column) are used in the manuscript:

ACF	autocorrelation function	*Sa*	arithmetic mean height Sa, µm
*D*_de_	the depth (height) of the dimples (valleys)	*Sal*	autocorrelation length, mm
*D*_di_	the diameter (width) of the dimples (valleys)	*Sda*	mean dale area, mm^2^
*D*_ds_	dimple-to-dimple distance	*Sdq*	root mean square gradient
*D*_dte_	dimple-to-edge distance	*Sdr*	developed interfacial areal ratio, %
GF	Gaussian filter	*Sdv*	mean dale volume, mm^3^
ITF	Instrument Transfer Function	*Sk*	core roughness depth, µm
MAF	moving average filter	*Sku*	kurtosis
MF	median filter	*Smc*	inverse areal material ratio, µm
MS	measurement speed (MS-1, MS-2, ..., MS-10)	*Smr*	areal material ratio, %
NS	noise surface	*Sp*	maximum peak height, µm
NSP	noise-sensitive parameters	*Spc*	arithmetic mean peak curvature, 1/mm
OTF	Optical Transfer Function	*Spd*	peak density, 1/mm^2^
*P*_Coef_	parameter coefficient	*Spk*	reduced summit height, µm
*P*_DiffCoef_	parameter difference coefficient	*Sq*	root mean square height, µm
PSD	power spectral density	*Ssk*	skewness
SDP	standard deviation plane	*Std*	texture direction, °
SEC	surface emptiness coefficient, calculated as *Sp*/*Sz*	*Str*	texture parameter
SWLI	Scanning White-Light Interferometry	*Sv*	maximum valley depth, µm
*W*_Cf_	Coiflet wavelet filter	*Svk*	reduced valley depth, µm
*W*_Dbn_	Daubechies wavelet filter of n-th order	*Sxp*	extreme peak height, µm
WNEP	wavelet noise extraction procedure	*Sz*	the maximum height of the surface, µm
WLI	White Light Interference		
*W*_RB_	reverse biorthogonal wavelet filter		
